# Game Theory, Conditional Preferences, and Social Influence

**DOI:** 10.1371/journal.pone.0056751

**Published:** 2013-02-25

**Authors:** Wynn C. Stirling, Teppo Felin

**Affiliations:** 1 Electrical and Computer Engineering Department, Brigham Young University, Provo, Utah, United States of America; 2 Marriott School, Brigham Young University, Provo, Utah, United States of America; Hungarian Academy of Sciences, Hungary

## Abstract

Neoclassical noncooperative game theory is based on a simple, yet powerful synthesis of mathematical and logical concepts: unconditional and immutable preference orderings and individual rationality. Although this structure has proven useful for characterizing competitive multi-player behavior, its applicability to scenarios involving complex social relationships is problematic. In this paper we directly address this limitation by the introduction of a conditional preference structure that permits players to modulate their preference orderings as functions of the preferences of other players. Embedding this expanded preference structure in a formal and graphical framework provides a systematic approach for characterizing a complex society. The result is an influence network that allows conditional preferences to propagate through the community, resulting in an emergent social model which characterizes all of the social relationships that exist and which leads to solution concepts that account for both group and individual interests. The Ultimatum game is presented as an example of how social influence can be modeled with conditional preferences.

## Introduction

The fundamental doctrine of game theory is that players make choices on the basis of preferences that take into account all factors that can influence their behavior. Classical game theory requires that preference orderings be *categorical*, meaning that they are individual, unconditional, and immutable. Arrow [1, p.51] put it succinctly: “It is assumed that each individual in the community has a definite ordering of all conceivable social states, in terms of their desirability to him. … It is simply assumed that the individual orders all social states by whatever standards he deems relevant.” With this model, each player comes to the game with a single preference ordering that, at least ostensibly, corresponds to its assessment of what is best for itself. Consequently, the natural concept of rational behavior is *individual rationality*: each player acts in a way that achieves its best possible outcome, regardless of the effect doing so has on other players. This doctrine is articulated by Harsanyi [2, p.13]: “Because all values and objectives in which the players are interested have been incorporated into their payoff functions, our formal analysis of any given game must be based on the assumption that each player has only one interest in the game—to maximize his own payoff.”

One of the consequences of the categorical preference ordering structure is that the game is stripped of social context. Indeed, this is often viewed as a strength of game theory, which is designed to remove all irrelevant and redundant issues and reduce the problem to its bare-bones mathematical essence. This modeling assumption is compatible with the “hourglass” approach described by Slatkin [3]: A complex problem is reduced to a tractable mathematical model by eliminating all irrelevant issues and, once a solution is obtained, it is expanded back into the original context for interpretation. Friedman [4, p.13] asserts that the context has very little to do with the way the game is solved: “The economist has little to say about the formation of wants; this is the province of the psychologist. The economist's task is to trace the consequences of any given set of wants.” According to this point of view, each player must come to the game with a categorical preference ordering that completely defines its personal tastes and values in a way that simultaneously accounts for such dissimilar motives as egoism, altruism, benevolence, malevolence, and indifference to the welfare of others, and which is not susceptible to change as a result of social interaction. Furthermore, once the preferences are defined, the process used to define them has no bearing on the way the game should be played.

This division of labor has been effective, particularly in economic settings where competition and market-driven forces dominate. But in more general social settings, expanding back into the context can lead to contradictions between observed and predicted behavior. Arrow [5, p.203] clearly delimits conditions for this division of labor approach to be valid. “Rationality in application is not merely a property of the individual. Its useful and powerful implications derive from the conjunction of individual rationality and other basic concepts of neoclassical theory—equilibrium, competition, and completeness of markets. … When these assumptions fail, the very concept of [individual] rationality becomes threatened, because perceptions of others and, in particular, their rationality become part of one's own rationality.” Thus, in contrast to Friedman's division of labor model, which separates the search for a rational solution from the context that generates the preferences, Arrow argues that the context can influence the rationality and, hence, the solution.

When an individual's concerns truly extend beyond it's own narrow interests, requiring it to express complex social interests within a framework that is explicitly designed to account for, and only for, individual interests is an artificial and unnecessary constraint. Although one may construct sophisticated and clever devices to cast social concerns as manifestations of individual interest, such attempts can lead to paradoxes such as having purely selfish reasons for acting unselfishly. At the end of the day, such mechanisms only allow the individual to simulate the interests of others; they do not allow general expressions of true social interest. Thus, although the dual premise of categorical utilities and individual rationality offers a convenient framework within which to model many decision making scenarios, it has its limitations. As Shubik [6, p.4] bluntly put it,

Economic man, operations research man and the game theory player were all gross simplifications. They were invented for conceptual simplicity and computational convenience in models loaded with implicit or explicit assumptions of symmetry, continuity, and fungibility in order to allow us (especially in a pre-computer world) to utilize the methods of calculus and analysis. Reality was placed on a bed of Procrustes to enable us to utilize the mathematical techniques available.

One way to make the bed a better fit for its occupant is to acknowledge that context matters when both defining and using preference orderings and to respond to the argument advanced by Hausman [7, p.136] for “the need to supplement game theory with systematic inquiry into how agents confronting a strategic interaction construct the game they play.”

The issue of how to account for social relationships has of course been raised by many others [8]. For example, behavioral game theory introduces greater psychological realism into the structure of payoffs by focusing on such factors as fairness and reciprocity [9]–[15]. Furthermore, repeated and evolutionary game theories provide frameworks by which players may learn or evolve behavior, such as cooperation, that conforms to the social context [16]–[19]. Such models are used to demonstrate that players are not exclusively motivated by narrow self-interest, but also care about the payoffs and intentions of others [20]–[21]. However, for the most part, the payoffs associated with these approaches are explicitly categorical, and any sociality generated by these models remains a function of individual interests.

Previous research on various forms of *conditional* preferences has included work that makes some distinctions between private and social preferences [22]–[25]. Conditional preferences are also used by multicriterion decision theory to characterize dependency relationships among different attributes [26]–[30]. Yet others have powerfully modified game theoretic assumptions by highlighting how the strategies of players are conditional on such factors as who focal players are surrounded by and the associated spatial distribution of strategies. For example, Szolnoki et al. use the ultimatum game to highlight the emergence of spatial patterns of empathy and fairness [31]–[32], along with highlighting how the imitation of emotions, rather than strategies leades to higher social welfare [33]. And finally, yet others have highlighted how social interaction itself can be conditional on the reputation of agents [34]. Questions of aggregation have of course also been central in fields such as strategy and organization theory [35]. All of these approaches represent important advances to our understanding of human and social interaction, and modifications to the strong assumptions made by traditional approaches in economics that assume categorical preferences.

However, our approach differs in substance, syntax and application from the above work in that we seek to develop a formal approach to conditional preferences, where extant social relations and ties give rise to both individual and social preferences. Building on the work of Stirling [36], we initiate a systematic inquiry by moving the study of preference formulation further upstream, though not necessarily to the psychological and sociological headwaters of preference origination. More modestly, the goal is to provide a mathematical framework within which such issues can be systematically studied. In short, *our explicit purpose and interest in this paper is to develop a formal model of how extant social relations, modeled as conditional preferences, play a role in games, social interaction, and aggregation*.

## Results

### Conditional preferences

We restrict attention to finite, strategic (normal form), noncooperative games. Let 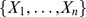, 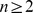, denote a set of players, and let 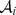 denote a finite space of feasible actions from which 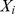 may choose one element. A *profile* is an array 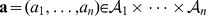. Under classical game theoretic assumptions, each 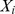 possesses a categorical utility 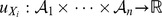. By its construction, a categorical utility naturally leads to solution concepts that require each 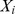 to choose an action such that the resulting outcome is maximally preferable to it, regardless of the effect the outcome has on other players. When making decisions in a social environment, however, it is natural for an individual to take into consideration the opinions of others when forming her own opinions. In short, individuals may be influenced by the preferences of others for a number of reasons: they may like (or dislike, for that matter) the others involved, they may value others' opinions, or they may have an existing relationship with others (familial, friendship or professional). Our approach is to incorporate these extended interests into the game by endowing each player with a family of *conditional utilities* that enable it to account for the social influence that the preferences of other players have on its preferences. Conditional utilities provide social linkages among players that enable simultaneous consideration of both individual and social interests. We show how graph theory can be used to characterize the way preference relationships propagate through a collective to generate an emergent social model that characterizes the interdependence relationships that exist and which leads to solution concepts that account for both group and individual interests. Our framework and formal model is general and thus it can readily be applied to a wide range of potential social contexts that feature extant social relations and influence.

To illustrate how conditional preferences provide a natural way to account for social relations and influence, consider a hierarchical organization such as a manager-employee scenario. The manager can choose either action 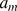 or action 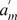, and the employee can choose either actions 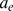 or action 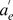. Under classical theory, each must determine their categorical preference ordering over the outcome space 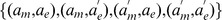. The priorities of the employee, however, are likely to be influenced by the priorities of the manager. One way to proceed is for the employee to reason as follows: If the most preferred outcome for the manager were, say, 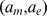, then the employee could define his ordering given that hypothesis. But if the manager were to most prefer 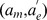, the employee would define a different ordering. Continuing, the employee could form a set of four different preference orderings, each one conditioned on a different hypothesized preference ordering by the manager. This could be done without the employee knowing the manager's actual preference ordering. The conditional preference orderings for the employee are the consequents of hypothetical propositions whose antecedents are assumptions regarding the preferences of the manager.

There is an important difference in the interpretation of the manager's preference ordering and the employee's preference orderings. Whereas the manager categorically orders her preferences over the possible joint actions of the two players, the employee conditionally orders his preferences for joint action *with respect to the preferences for joint action of his manager*. Thus conditional preferences can provide a powerful approach to more formally modeling how extant social relations play a role in influencing the behavior of actors.

This line of reasoning is similar to the type of reasoning employed by multivariate probability theory. The power of probability theory is succinctly expressed by Shafer (cited in [37, p.15]): “probability is not really about numbers; it is about the structure of reasoning.” And one of the powerful reasoning structures that probability theory offers is a framework within which to form hypothetical propositions. In the probabilistic context, given a collective of two discrete random variables 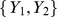, the conditional probability mass function 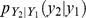 is the consequent of a hypothetical proposition regarding the probability that 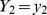, given the antecedent that 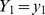. This reasoning structure is epistemological, both semantically and syntactically. The semantics deals with notions of knowledge, what to believe, and how to justify beliefs, and the syntax deals with the way beliefs are expressed and combined. For example, the chain rule, 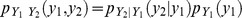, governs the way beliefs in one domain, as expressed by 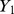
's marginal probability mass function, should be combined with conditional beliefs in another domain, as expressed by 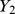
's conditional probability mass function, to govern the beliefs of the collective.

This reasoning structure, however, is not limited to epistemological contexts; it may also be applied to contexts where the semantics deals with notions of effective and efficient action, and where the syntax deals with the way preferences for taking action are expressed and combined. Given a set 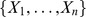 with outcome space 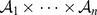, let the *parent set* 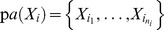 be the 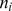-element subset whose preferences influence 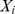
's preferences. Now consider the hypothetical proposition whose antecedent is the assumption that 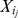 considers, for whatever reason, 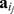 to be the outcome that should occur. We term such a profile a *conjecture*. Let 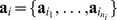 denote the *joint conjecture* of 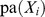. The consequent of this hypothetical proposition is a *conditional utility* 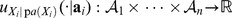 for each 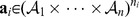. (This notation is analogous to the notation used for conditional probability. The argument on the left side of the conditioning symbol “
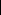
” denotes the profile corresponding to 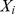, and the argument on the right side of the conditioning symbol denotes the joint conjecture of the agents who influence 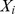.) If 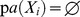, then 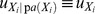, a categorical utility. Without loss of generality (via a positive affine transformation if necessary), we may assume that all utilities are non-negative and sum to unity. With this constraint, the utilities possess the syntax of a mass function. The collective 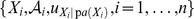 constitutes a finite, normal form, noncooperative *conditional game*.

### Aggregation

Neoclassical game theory has not developed in a way that sanctions notions of a group-level preference ordering. As [38, p.124] has observed, “It may be meaningful, in a given setting, to say that group ‘chooses’ or ‘decides’ something. It is rather less likely to be meaningful to say that the group ‘wants’ or ‘prefers’ something.” Although this sentiment may be appropriate when all utilities are categorical, this line of reasoning loses much of its force when social linkages exist among the players. The existence of conditional utilities enables an important added dimension to decision making in a complex social environment: Once interest extends beyond the self, considerations of group-level interest become relevant and should not be suppressed. Returning to the manager-employee example, a natural question is: How should the categorical preferences of the manager and the conditional preferences of the employee be combined to form an emergent preference ordering for the group?

One way to address these questions is to exploit the obvious syntactical similarity of conditional utilities and conditional probabilities by forming an analogy between probability theory and utility theory. If this were done, then mathematical operations such as marginalization, independence, and the chain rule could provide a powerful framework within which to characterize and analyze complex social systems. It is not sufficient, however, simply to make a syntactical correspondence between belief modeling and preference modeling. Beliefs are not preferences (unless we engage in wishful thinking), and considerations of what to believe are not the same as considerations of what to prefer. Thus it is not obvious that the syntax of probability theory will apply in a preference-oriented context. Any such correspondence must be rigorously justified.

To address this issue, it is helpful first to take a close look at the way probability theory is developed. The traditional treatment is to view conditional probability as the ratio of a joint probability and a marginal probability; i.e., 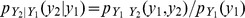, from which the chain rule follows trivially. The important feature of this development, however, is that the definition of conditional probability is dependent on the epistemological context; it is a re-normalizing of probability: 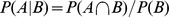; that is, the belief that 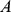 is true given that 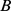 is true is the ratio of the belief that both are true and the belief that 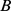 is true. To proceed along these lines to justify the chain rule in a preference-oriented context would either require a) constructing an analogue to a probability space, which would seem to be a rather tedious undertaking, or b) ignoring foundational theoretical concerns and relying on intuition and *ad hoc* reasoning.

Fortunately, there is a way to arrive at the chain rule that does not rely upon the standard definition of conditional probability. This approach requires an important change in perspective. Rather than view the joint probability mass function as the primary component from which marginal and conditional probabilities can be derived, an alternative view is to consider the conditional and marginal probabilities as the primitive components from which the joint probability can be synthesized. The development of probability theory from this perspective has been provided by [39] and [40], and is entirely in keeping with the observation by [37] that dependence relationships are the fundamental building blocks of probabilistic knowledge.

**Figure 1:**
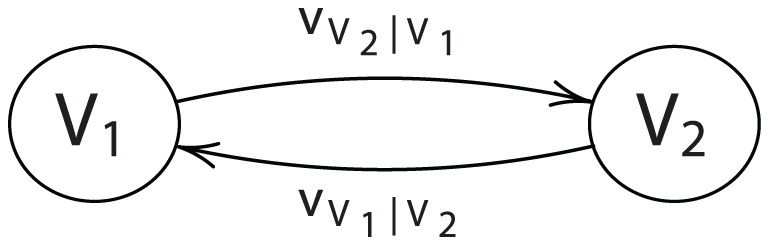
A two-player cyclic influence diagram.

Motivated by the probability context, let us consider a general context scenario involving a collective of entities, each of which possesses some notion of ordering the alternatives available to it, and whose ordering notions can be influenced by other agents. A convenient and powerful way to express such influence is with graph theory. Consider the two-vertex graph illustrated in Figure 1, where 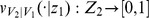 for each 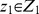, and 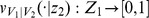 for each 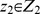 be conditional ordering functions for 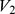 given 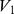 and 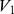 given 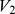, respectively, for some finite domains 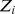, 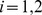. Also, let 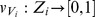 be categorical ordering functions for 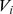, 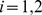.

**Figure 2:**
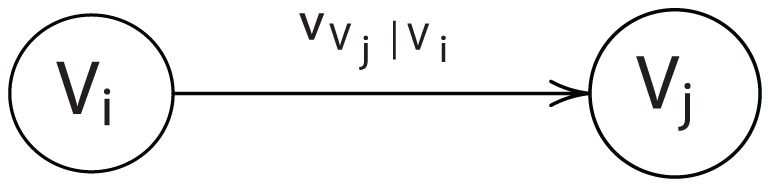
A two-player acyclic influence diagram.

Suppose we wish to synthesize a joint ordering function 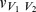 from these components. The general form would be
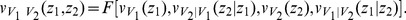
(1)This form permits the possibility of indirect self-influence, that is, 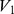 influences 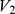 which in turn influences 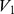, and so forth, which could lead to an infinite regress. We may eliminate such behavior by stipulating that influence flows must be uni-directional, that is, by imposing acyclicity. Then we may simplify the structure by eliminating one set of marginal and conditional orderings from the argument list. If this is done, however, consistency requires that the joint ordering be invariant to the way the problem is framed, that is, we require
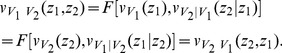
(2)Framing invariance is a strong condition to impose upon a collective. It means that the same joint ordering will obtain for setting 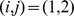 as for setting 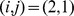 in Figure 2.

More generally, framing invariance means that if aggregation can be framed in more than one way using exactly the same information (although coded differently), then all framings will result in the same joint ordering. Framing invariance is always assumed in probabilistic contexts, since the joint probability mass function is invariant to the order in which the random variables are considered. Given a set of random variables , let 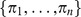 be an arbitrary permutation of 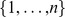. Then 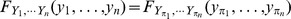 must hold.

In an arbitrary context, however, framing invariance cannot be automatically assumed to hold. Consider the manager-employee scenario. Under the original framing, the manager possesses a categorical utility 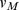 and the employee possesses a family of conditional utilities 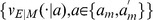. Framing invariance requires that a categorical utility 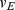 must exist for the employee and a family of conditional utilities 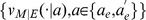 must exist for the manager such that
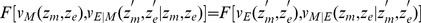
(3)for all 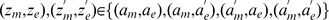. For this to hold, some concept of reciprocity or symmetry must exist between the two participants. Obviously, framing invariance would fail if the manager were so intransigent that she would not, under any circumstances, take the opinions of the employee under consideration, or if the employee were so incompetent that he could not form preferences over the outcome space. Invoking framing invariance does not require that the alternate framing actually be defined, only that it exist in principle. The richness and variability of human behavior, however, make it impossible to impose this condition without justification. Nevertheless, framing invariance provides a reasonable framework within which to model many social relationships, especially for scenarios involving coordinated behavior or the need to compromise, and represents a significant generalization to the traditional categorical utility model. Obviously, the utilities of classical game theory are framing invariant, since then the conditional utilities coincide with the categorical utilities. Thus, framing invariance is a weaker condition than the categorical assumption.

In addition to acyclicity and framing invariance, we must impose one more condition on 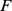. Referring to (2), suppose 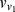 were increased but 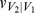 were held constant. Common sense dictates that 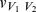 should not decrease. A similar argument applies of 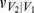 is increased and 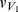 is held constant. Thus, 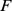 must be non-decreasing (monotonic) in each argument in order to avoid counter-intuitive influence behavior.

**Figure 3:**
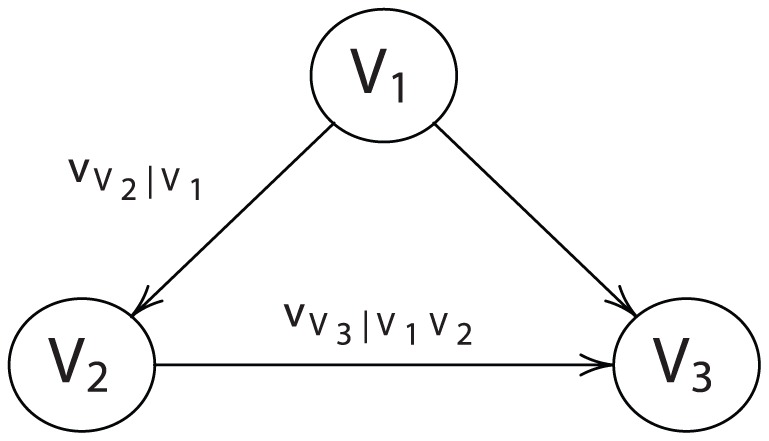
A three-vertex DAG.

For collectives involving more than two entities, we must move beyond the simple graph defined in Figure 2. In general, a *directed graph* 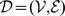 is a collection of *vertices* 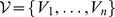 and *directed edges* 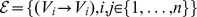 that constitute the links between vertices. Each 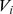 takes values over some finite domain space 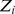. A *directed acyclic graph* (DAG) is a directed graph such that no sequence of edges returns on itself. If a vertex has no incoming edges (i.e., it has no parents), it is a *root vertex*. To complete the specification of a DAG, each root vertex must possess a categorical preference ordering over its own states. Figure 3 illustrates a three-vertex DAG with 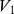 (a root vertex) influencing 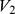, and with 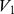 and 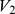 both influencing 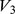. The edges are denoted by the conditional ordering functions 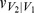 and 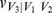.

The DAG structure is not dependent on any specific context; it is a general model of how influence propagates uni-directionally through any kind of collective. (Graphical models of complex economic systems have recently appeared in the literature as a convenient and powerful means of representing social relationships (see [41]–[44]). Such models are often used to characterize the spread of infectious diseases and the propagation of information. However, none of these discussions involve the formal modeling of conditional preferences.) A key issue is how the individual preferences, as represented by categorical preference orderings for root vertices and conditional preference orderings for children vertices, can be combined to create a preference ordering for the group. The main contribution of this paper is the *aggregation theorem*, which is formally stated and proved in the Methods and Materials section. Essentially, the aggregation theorem establishes conditions that justify applying the chain rule syntax to preference aggregation semantics. We apply this result to the social influence context as follows.

Let 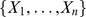 be an 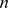-member influence network such that each 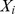 possesses its own finite action space 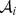 and orders its preferences over the outcome space 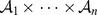. Let 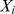
's parent set, denoted 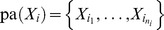 be the 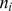-element subset whose preferences influence 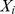
's preferences. If 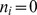, then 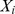 is a root vertex and 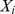 will possess a categorical utility over the outcome space. If 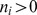, then 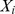
's conditional utility will be of the form 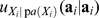, where 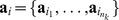, the set of conjectures of 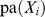. Applying the aggregation theorem,
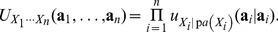
(4)


The aggregation theorem establishes that acyclicity, monotonicity, and framing invariance are necessary and sufficient for the chain rule to apply. Thus, given these conditions, the syntax of probability theory applies to preference modeling as well as to the conventional application of the chain rule to belief modeling. However, simply imposing the chain rule on a set of conditional preference orderings without complying with these requirements would be problematic.

### Interpretations

The aggregated utility is a very complex function and interpreting it is equally complex. Analogous to the way a joint probability mass function provides a complete description of the dependency relationships that exist among the random variables in terms of belief, the aggregated utility provides a complete description of the dependency relationships that exist among the players in terms of preference. Interpreting the aggregated utility, however, is not as straightforward as interpreting a joint probability mass function. To develop this concept, we introduce the notion of concordance and then provide interpretations of independence and marginalization.

#### Concordance

Social influence can propagate through a group in complicated ways. 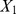 may influence 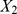, who may in turn influence 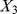, and so on, thereby creating a cascade of social relationships that interconnect the players in ways that cannot be easily predicted. In this expanded context, it is not sufficient simply to create a payoff array to be subjected to standard solution concepts such as dominance and equilibrium. Instead, we must construct a social model that accounts for all of the interrelationships. One concept that applies in multiple contexts is the notion of *concordance*, and in the sequel we will term 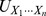 a *concordant utility*.

Since it is a function of 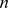 profiles, the concordant utility cannot be used directly to define a group-level ordering over the outcomes. Rather, it provides a representation of the social consistency of the group, in that it provides a measure of the degree of severity of controversy. To illustrate, let us consider a two-agent group 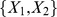. Le 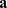 and 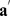 be such that 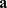 is best for 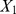 and next-best for 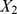, and 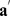 is worst for 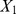 and best for 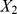. It is reasonable to argue that if both were to conjecture 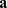, the degree of controversy would be fairly small, since both agents receive a reasonable reward. If both were to conjecture 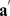, however, the outcome would be worst for one and best for the other; hence the degree of controversy would be quite large. Accordingly, the condition 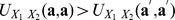 would obtain.

The concordant utility permits the definition of an emergent notion of social consistency, namely, an aversion to controversy. The expresson 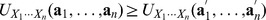 means that if the group were jointly to conjecture 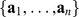, the level of controversy for 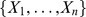 would be less than or equal to what it would be if the group were jointly to conjecture 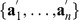. “Consistency,” as considered here, can be positive, in the sense of cooperation for individuals with common interests, or negative, when conflict would not be controversial, as would be the case in military operations or athletic competitions. In general, concordance captures the context of the game as the conditional preferences propagate through the vertices of the network.

#### Independence

Let 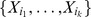 and 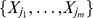 be disjoint subgroups such that
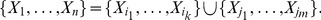
(5)These subgroups are *independent* if neither subgroup influences the other, in which case the concordant utility of the union is the product of the concordant utilities of the subgroups. That is,
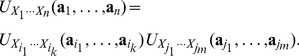
(6)


#### Marginalization

The concordant utility provides a complete representation of the way preferential influence, as modeled by *ex ante* conditional utilities, propagates through a collective of players. Marginalization extracts *ex post* preferences of each player as a result of this propagation:
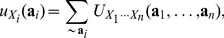
(7)where the notation 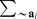 means that the sum is taken over all arguments except 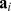. Marginalization is the mechanism by which individual preferences emerge as a result of the social relationships that exist among individuals. Thus, even though an *ex ante* categorical ordering may not be given, marginalization provides an *ex post* unconditional ordering; that is, after consideration of the social relationships among the agents have been taken into account. These *ex post* categorical utilities represent the players' enlightened self-interest after systematically taking into account the degrees to which they are influenced by the preferences of others as expressed by their *ex ante* conditional utilities.

**Example 1** *Let* 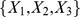 *comprise a three-player collective such that* 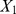 *possesses a categorical utility* 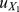, 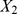 *possesses a conditional utility* 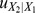, *and* 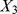 *possesses a conditional utility* 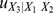 *(see* *Figure 3* *). Applying the chain rule yields the concordant utility*

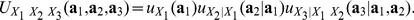
(8)
*Since* 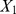 *possesses an ex ante categorical utility, the ex post utility for* 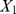 *will coincide with that categorical utility. The* ex post *utility of* 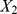 *is given by*

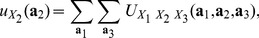
(9)
*with a similar expression for the* ex post *utility for* 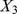.

### Social solution concepts

The *ex post* marginal utilities defined by (7) provide a preference ordering for individual players. Once obtained, the history of their creation ceases to be relevant. In fact, such a procedure is nothing more than an application of Friedman's division of labor. They are unconditional and are indistinguishable in structure from *ex ante* categorical utilities. Consequently, they may be used according to any classical solution concept, such as Nash equilibria. If this were the end of the story, then all of the above development would be nothing more than a prelude to classical game theory, and we would fall short of our goal to offer a true expansion to the theory. But there is more to be said. In contrast to classical game theory, which eschews notions of group-level preferences, the existence of explicitly defined social influence relationships opens the possibility of defining a group-level preference ordering that is more than just an aggregation of categorical preference orderings.

Our approach is to construct another kind of marginal. Just as we may extract marginals from the concordant utility for each individual, we may also extract a marginal for the group. To proceed, we observe that since each player can control only its own actions, what is of interest is the utility of all players *making conjectures over their own action spaces*.

**Definition 1** *Consider the concordant utility* 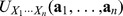. *Let* 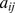 *denote the* 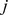 *th element of* 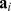 *; that is,* 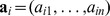 *is* 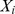 *'s conjecture profile. Next, form the action profile* 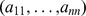 *by taking the* 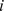 *-th element of each* 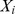 *'s conjecture profile,* 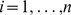. *Now let us sum the concordant utility over all elements of each* 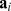 *except* 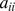 *to form the group utility* 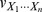 *for* 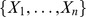, *yielding*

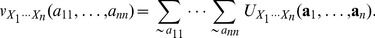
(10)


The group utility provides a complete *ex post* description of the social relationships between the members of a multiplayer group. Unless its members are independent, this utility is not simply an aggregation of categorical utilities. Rather, it is an emergent notion of group preference.

Although the group does not act as a single entity, or superplayer, the group utility nevertheless informs each member of the group regarding the effect of their collective actions on the society. Each member can extract its own single-player utility as a function of its own action by computing its own marginal welfare function.

**Definition 2** *The* individual utility 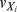 *of* 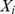 *is the* 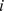 *th marginal of* 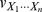, *that is*,
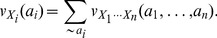
(11)


The existence of both group-level and individual-level preference orderings provides a framework within which to create solution concepts that simultaneously take into consideration the (emergent) interests of the group and the individuals. We describe one such concept that involves negotiation.

**Definition 3** *The* maximum group welfare *solution is*

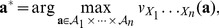
(12)
*and the* maximum individual welfare *solution is*

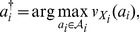
(13)


If 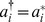 for all 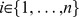, the action profile is a *consensus* choice. In general, however, a consensus will not obtain, and negotiation may be required to reach a compromise solution.

The existence of group and individual utilities provides a rational basis for meaningful negotiations; namely, that any compromise solution must at least provide each player with its security level—that is, the maximum amount of benefit it could receive regardless of the decisions that others might make. The security level for 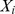 is the maximin profile, defined as
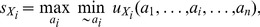
(14)where 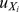 is the *ex post* utility given by (7).

In addition to individual benefit, we must also consider benefit to the group. Although a security level, per se, for the group cannot be defined in terms of a minimum guaranteed benefit (after all, the group itself does not actually make a choice), a possible rationale for minimum acceptable group benefit is that it should never be less than the smallest benefit to the individuals. This approach is consistent with the principles of justice espoused by [45], who argues, essentially, that a society as a whole cannot be better off than its least advantaged member. Accordingly, let us define a security level for the group as 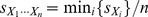, where we divide by the number of players since the utility for the group involves 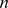 players.

Now define the *group negotiation set*

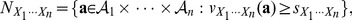
(15)the *individual negotiation sets*

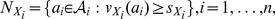
(16)and the *negotiation rectangle*

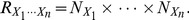
(17)The negotiation rectangle is the set of profiles such that each member's element provides it with at least its security level. Finally, we define the *compromise set*

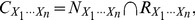
(18)which simultaneously provides each member of the group at least its security level, as well as meeting the group's security level. If 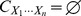, then no compromise is possible at the stated security levels. One way to overcome this impasse is to decrement the security level of the group iteratively by a small amount, thereby enlarging 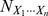 until 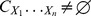. If 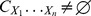 after the maximum reduction in group security has been reached, then no compromise is possible, and the group may be considered dysfunctional. Another way to negotiate is for individual members to iteratively decrement their security levels.

Once 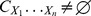, any element of this set provides each member, as well as the group, with at least its security level. If 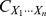 contains multiple elements, then a tie must be broken. One possible tie-breaker is
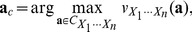
(19)which provides the maximum benefit to the group such that each of its members achieves at least its security level.

### Sociation

This development has assumed the full generality of conditioning; namely, that a conditional utility depends on the entire conjecture profiles of all of the parents, and that the conditional utility is a function of all elements of the action profile. This fully general model can be extremely complex, since each player is under obligation to define its preferences for every possible joint conjecture of its parents—a potentially intractable task. Although it is necessary for the theory to have the ability to accommodate maximal complexity, sociation provides a way to control complexity in keeping with the observation by [46, p.176] that “complexity is no argument against a theoretical approach if the complexity arises not out of the theory itself but out of the material which any theory ought to handle.” It can be the case that a player does not condition its preferences on the entire conjecture profiles of its parents. It can also be the case that a player's utility does not depend upon the entire action profile. To account for such situations, we introduce the notion of *sociation*.

Suppose 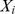 has 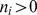 parents 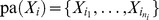, with conditional utility 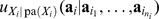, where the joint conjecture 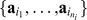 comprises the conjectures of 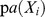.

**Definition 4** *A* conjecture subprofile *for* 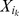, *denoted* 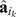, *is the subset of* 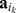 *that influences* 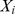 *'s conditional utility. We then have*

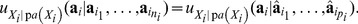
(20)

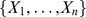
 *is* completely conjecture sociated *if* 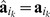 for 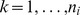 and 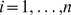.

*It is* completely conjecture dissociated *if* 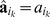 for 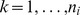 and 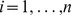, *in which case*,
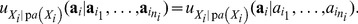
(21)
*Otherwise, the group is* partially conjecture dissociated.

**Definition 5** *A* utility subprofile, *denoted* 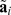, *comprises the subset of actions by* 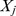, 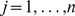, *that affect* 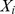 *'s utility. We then have*

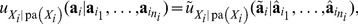
(22)
*where* 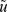 *denotes* 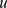 *with the dissociated elements of its argument removed*. 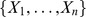 is completely *utility sociated* if 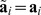 for 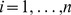. It is completely utility dissociated *if* 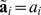 for 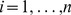, in which case
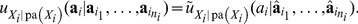
(23)Otherwise, the group is *partially utility dissociated*.

For a partially sociated group, the concordant utility assumes the form
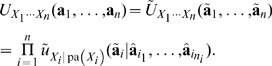
where 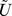 is 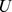 with the dissociated arguments removed.

**Definition 6** *A group* 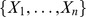 *is completely dissociated if it is both completely conjecture dissociated and completely utility dissociated, in which case*,
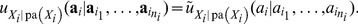
(24)


For a completely dissociated group, the concordant utility becomes the group utility.
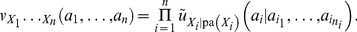
(25)


## Discussion

### The ultimatum game

The Ultimatum game ([47]) has received a great deal of attention as an example of situations where experimental evidence contradicts the assumptions of classical game theory. Ultimatum is a two-player game defined as follows: 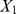, the *proposer*, has access to a fortune, 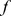, and offers 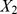, the *responder*, a portion 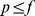, and 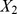 chooses whether or not to accept the offer. If 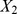 accepts, then the two players divide the fortune between themselves according to the agreed upon portions, but if 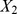 declines the offer, neither player receives anything. The game loses little of its effect, and its analysis is much simpler if we follow the lead of [48], and consider the so-called minigame, with only two alternatives for the proposer: 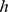 and 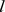 (high and low), with 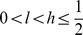. This minigame analysis captures the essential features of the continuum game and permits us to see more clearly the relationships between the two players. With this restriction, the action sets for the two players are 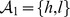 and 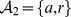 for 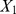 and 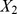, respectively.

The payoff matrix for the Ultimatum minigame is illustrated in Table 1. This game has a dominant strategy for each player; namely, 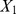 should play 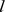 and 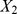 should play 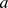. The response of many players, however, indicates that they typically are *not* utility maximizers. Thus, this game is an excellent example of a situation where social considerations appear to be significant. Analysts of the game have theorized that the responders decline an offer they deem to be unfair because they are emotionally connected with the consequence. A term that seems to capture this emotion is *indignation*. Another feature that emerges from the play of this game is that the proposer may be motivated by considerations other than greed. Even if the proposer is greedy, it may still make an equitable offer if it suspects that the responder would be prone to reject an inequitable one. A concept that expresses this emotion is *intemperance*. We denote these two emotional attributes by the intemperance index 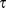 and the indignation index 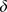, and assume that 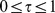 and 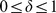. The condition 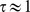 means that the proposer is extremely avaricious, but if 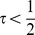, then the proposer may be viewed as having altruistic tendencies. The condition 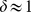 means that the responder is easily offended, while 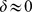 means that it is extremely tolerant.

**Table 1:** The payoff matrix for the Ultimatum minigame.

	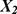	
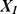	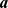	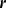
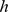	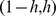	(0, 0)
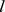	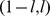	(0, 0)

With conventional game, the payoffs define their utilities, but when social issues are involved, we need to relax the requirement for strict alignment of preferences and payoffs in order to apply the social parameters. There are many ways to frame such a game; one natural way is to endow the proposer 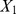 with a categorical utility and endow the responder 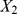 with a conditional utility.

The general fully sociated game would require the players to define preferences over the entire product space 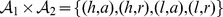, which would require 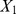 to specify four values for its categorical utility and 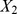 to specify sixteen values (four for each of 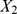
's four possible conjectures). The game may be simplified by adopting various levels of dissociation, and to keep this presentation as simple as possible, we will assume a condition of complete dissociation. Under this condition, each 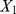
's categorical utility is with respect to its actions only, and 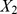
's conditional utility is with respect to its actions only, conditioned on conjectures regarding 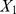
's actions only. We thus make the following assignments.
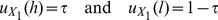
(26)

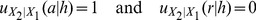
(27)

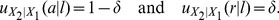
(28)Under the assumption of complete dissociation, the concordant utility reduces to the group utility (see (10)), with components of the form 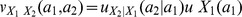 for 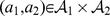 (see (35)), yielding
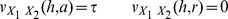


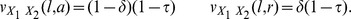



The outcome that maximizes group welfare depends on the values of 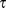 and 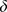 as follows:
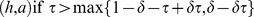
(29)

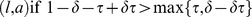
(30)

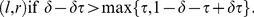
(31)


Upon simplification of these expressions, we may identify values of 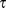 and 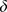 that maximize group welfare.
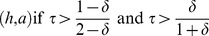
(32)

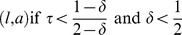
(33)

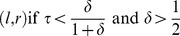
(34)Notice that the outcome 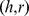 is never the maximum outcome for the group.

The individual welfare functions are (see (11)):
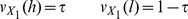


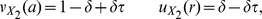
from which it is easily seen that 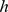 is best for 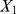 if 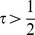 and 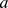 is best for 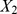 if 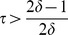.

**Figure 4:**
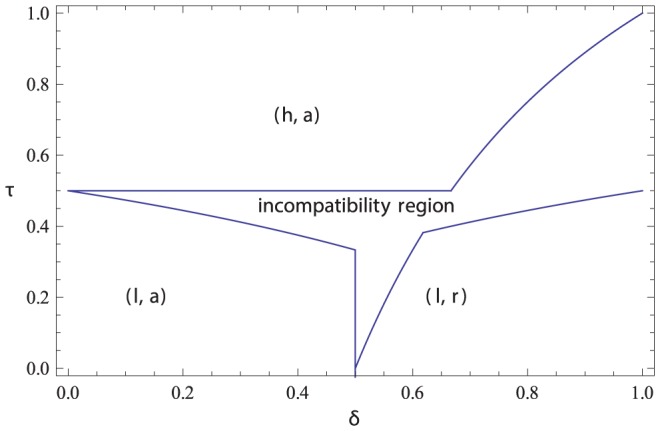
Cross-plot of intemperance (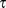) versus indignation (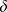) for group and individual preference compatibility.

Figure 4 displays the regions of the 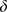-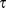 plane where the group and individual preferences are in agreement. The region labeled 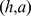 represents the values of 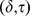 where the group 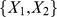 prefers the joint outcome 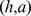 to all other outcomes and, simultaneously, 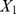 prefers 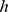 to 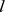 and 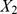 prefers 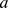 to 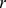. Similar interpretations apply to the regions labeled 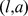 and 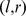. In all other portions of the 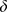-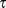 plane, group preferences are not consistent with individual preferences.

This example illustrates how social parameters may be explicitly embedded into the mathematical structure of the utilities. Without incorporating context into the model, deviations from behavior predicted by the payoffs would require the invocation of psychological and sociological attributes that, while not part of the mathematical model, are necessary to explain the deviations. They merely overlay the basic mathematical structure of a game and avoid or postpone a more profound solution, namely, the introduction of a model structure that explicitly accounts for complex social relationships and notions of rational behavior that extend beyond narrow self-interest and categorical preferences.

## Conclusion

In this paper we have developed a formal model of conditional preferences, with application to various forms of social interaction such as game theory and preference aggregation [36]. We modify traditional models of social interaction that presume categorical individual preferences, preferences that are assumed to be fixed and invariant. We provide a model of how extant social relations influence and condition individual preferences and aggregate, social outcomes. We have thus sought to introduce increased realism to existing models of games and social interaction by showing how individual preferences interact in nontrivial ways in social settings and how collective outcomes aggregate and emerge as a result of this conditioning, social interaction and influence.

Our formal approach is generalizable to many specific social contexts, beyond the stylized settings of game theory and social choice. That is, our approach is general in the sense that extant social relations and conditioning can feasibly be driven by many factors. Our model is applicable to any setting where agents are linked and some form of interaction or aggregation is needed. For example, the conditioning of individual preferences might have to do with such social or interactional factors as spatial relations, expertise, hierarchy, professional and managerial relationships, or friendship networks. Our model is generalizable to these settings and might thus also be applied to settings such as the design of artificial or expert systems, the aggregation of information, or coordination within, or design of, social systems.

Since our approach is meant to be general, future work might look at the boundaries of our argument, that is, how social conditioning perhaps differs between various social contexts and how our general model of conditioning and social influence may require context-specific amendments. Furthermore, our model also takes extant relations as a given and thus has little to say about where social relations or structures come from, or how these structures evolve as individuals interact over time. Thus there is an opportunity to study the emergence and evolution of the social relations that condition individual and aggregate preferences and outcomes.

Overall, many different solution concepts have been proposed since the inception of game theory, with the bulk of attention focusing on concepts that conform to the individual rationality assumption, including minimax theory, notions of equilibrium, coalition forming, and repeated games. For example, repeated and evolutionary game theories have provided frameworks within which to study how players learn and adapt to their social environments. The field of behavioral economics has sought to imbue games with greater psychological realism by introducing social parameters into the preference models. All of these threads, however, are ultimately connected to the fundamental mathematical structure of categorical utilities and the logical structure of individual rationality. This paper at least strains, if not breaks, those threads: categorical preference orderings are explicitly replaced by conditional preference orderings and individual rationality is replaced by a notion of simultaneous group and individual accommodation. We have thus developed a formal model of social interaction, focused on capturing how individuals influence each other and how this influence propagates in and through social structures, both at the individual and aggregate levels.

## Materials and Methods

### 

#### Theorem 1 The Aggregation Theorem

*Let* 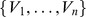 *be the vertices of a DAG, where each* 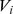 *takes values in some set* 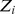. *Let* 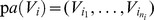 *be the set of* 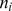 *parents of* 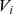. *For any vector of states* 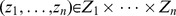 *t* 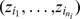 *denote the subvector corresponding to* 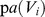. *Also, let* 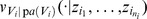 *be a mass function defining the conditional ordering of the states of* 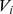 *given* 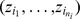. *If* 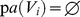, *then* 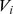 *is a root vertex and* 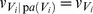, *a categorical ordering of the states of* 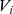. *The joint ordering function for the collective is of the form*

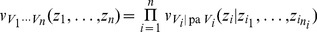
(35)if and only if acyclicity, monotonicity, and framing invariance hold.

We first prove this result for 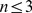 and then extend to the general case. Consider three-vertex DAG illustrated in Figure 3 and let 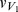 denote a mass function that orders the states of 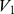. Let 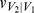 denote a mass function that conditionally orders the states of 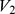 given the state of 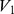, and let 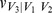 denote a mass function that conditionally orders the states of 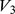 given the sates of 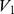 and 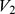.

One way to frame this problem is to aggregate 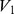 and 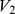 first and to aggregate the result with 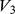, yielding
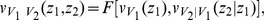
(36)

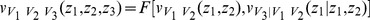
(37)

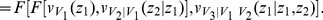
(38)


Another way to frame the problem is first to aggregate 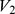 and 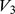 conditioned on 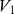 and then to aggregate the result with 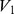, yielding
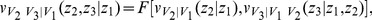
(39)

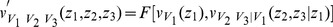
(40)

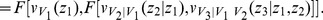
(41)


By framing invariance, these two ways of aggregating must yield the same joint ordering:
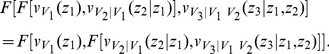
(42)Thus, the joint ordering satisfies the *associativity equation*

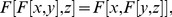
(43)evidently first studied by [49]. By inspection, setting 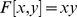 is a solution to this equation. Cox [39] (also see [40]) has shown that if 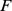 is differentiable, then the general solution to the associativity equation is of the form 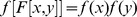 for any monotonic continuous 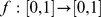. Taking 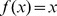 yields
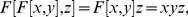
(44)hence,
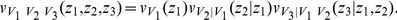
(45)


**Figure 5:**
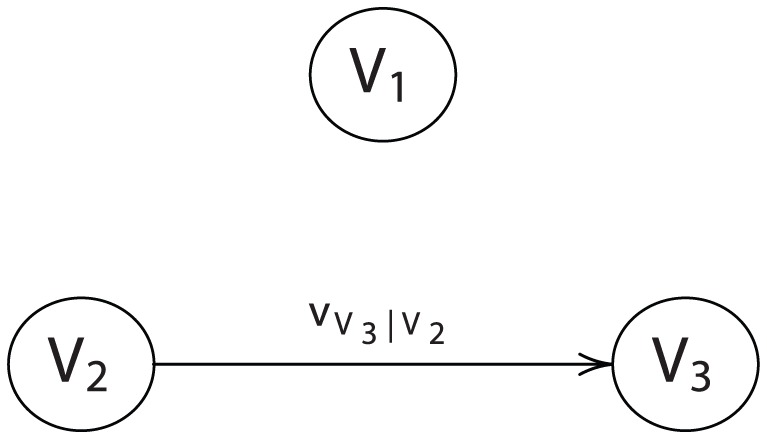
A two-vertex DAG with a dummy vertex.

To deal with the case 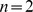, consider the DAG illustrated in Figure 5, where 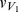 is a dummy vertex with a singleton range space 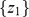, with 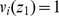. Since 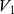 has no influence on either 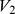 or 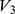, 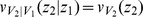 and 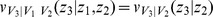. Substituting these expressions into (41) yields
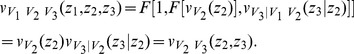
(46)


To establish this result for 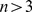, we apply the aggregation theorem to obtain
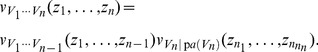
(47)But
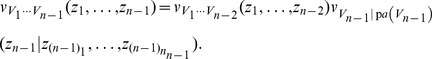
(48)Successive applications results in
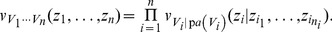
(49)which is (35).

To prove the converse, monotonicity and acyclicity are obvious. To establish framing invariance, we first consider the case 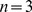. Applying (35),
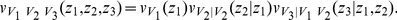
(50)We may recognize the first two terms on the right-hand side of (50) as the joint ordering function for 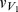 and 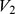, that is,
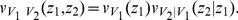
(51)Thus,
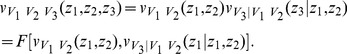
(52)


Furthermore, we may recognize the last two terms on the right-hand side of (50) as the conditional joint ordering function for 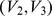 given 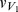, that is,
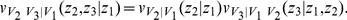
(53)Thus,
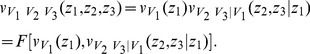
(54)Comparing (52) with (54) establishes framing invariance for 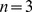. Then general case for 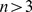 follows by similar manipulations of (35).
